# The risk profiles of pregnancy-related cerebral venous thrombosis: a retrospective study in a comprehensive hospital

**DOI:** 10.1186/s12883-024-03676-2

**Published:** 2024-05-31

**Authors:** Shaoying Wang, Ming Yao, Nan Hu, Juntao Liu, Bin Peng

**Affiliations:** 1 Department of Neurology, Peking Union Medical College Hospital, Peking Union Medical College and Chinese Academy of Medical Sciences, Beijing, 100730 China; 2 Department of Obstetrics and Gynecology, Peking Union Medical College Hospital, Peking Union Medical College & Chinese Academy of Medical Sciences, Beijing, China

**Keywords:** Cerebral venous thrombosis, Pregnancy, Risk factor, Thrombophilia, Antiphospholipid syndrome

## Abstract

**Objectives:**

To investigate the risk factors and underlying causes of pregnancy-related cerebral venous thrombosis (PCVT).

**Methods:**

A retrospective cohort of 16 patients diagnosed with CVT during pregnancy and postpartum (within six weeks after delivery) in a comprehensive hospital in China between 2009 and 2022 were carefully reviewed, focusing on demographic, clinical, and etiological characteristics, especially underlying causes. We matched 16 PCVT patients with 64 pregnant and puerperal women without PCVT to explore risk factors and clinical susceptibility to PCVT.

**Results:**

PCVT occurred commonly during the first trimester (43.75%) and the puerperium (37.5%). The frequency of anemia, thrombocytosis and thrombocytopenia during pregnancy, dehydration, and pre-pregnancy anemia was significantly higher in women with PCVT than in those without PCVT (*P* < 0.05). Among the 16 patients, five were diagnosed with antiphospholipid syndrome and one was diagnosed with systemic lupus erythematosus. Three patients had distinct protein S deficiency and one had protein C deficiency. Whole Exome Sequencing (WES) was performed for five patients and revealed likely pathogenic mutations associated with CVT, including heterozygous PROC c.1218G > A (p. Met406Ile), heterozygous PROS1 c.301C > T (p. Arg101Cys), composite heterozygous mutation in the F8 gene (c.144-1259C > T; c.6724G > A (p. Val2242Met)) and homozygous MTHFR c.677C > T (p. Ala222Val).

**Conclusions:**

The occurrence of anemia, thrombocytopenia and thrombocytosis during pregnancy, dehydration and pre-pregnancy anemia suggested a greater susceptibility to PCVT. For confirmed PCVT patients, autoimmune diseases, hereditary thrombophilia, and hematological disorders were common causes. Screening for potential etiologies should be paid more attention, as it has implications for treatment and long-term management.

## Introduction

Cerebral venous thrombosis (CVT) is a special type of cerebrovascular disease that results from impaired blood flow or cerebrospinal fluid circulation, leading to intracranial hypertension and focal brain damage [1]. CVT affects female more than male, and women of reproductive ages are common victims [2]. Pregnancy has been identified as a risk factor for CVT. The incidence of pregnancy-related CVT (PCVT) varies by country and region. In high-income countries, it is approximately 10/100,000 deliveries, accounting for 5–20% of all CVT cases. However, in countries such as India or Mexico, the incidence can be up to 10 times higher, with PCVT cases comprising as much as 60% of all CVT cases [3]. PCVT accounts for approximately 11–59% of female CVT patients [4]. In the International Study of Cerebral Venous Thrombosis (ISCVT), among 624 CVT cases, 12.3% were PCVT. Among 381 female CVT patients under 50 years old, 20.1% were PCVT, with 6.3% occurring during pregnancy and 13.8% occurring during the postpartum period [5]. In China, the incidence of PCVT is about 202 per 100,000 pregnancies, and the mortality rate is 11.63% [6], imposing a significant burden on pregnant women and their families.

CVT is associated with several risk factors, which can be classified into transient and permanent ones. [4] Among them, systematic diseases such as acquired thrombophilia like systemic lupus erythematosus (SLE) and hereditary thrombophilia have received increasing attention. However, most of the previous articles have been based on the overall CVT population. In addition, there is a misconception to attribute the cause of PCVT solely to pregnancy-induced hypercoagulability. Whether the same risk factors are established in pregnant women needs to be further investigated. Previous case reports or series show that anemia, obesity, smoking, cesarean section, epidural and spinal anesthesia during childbirth, pregnancy-induced hypertension, ovarian stimulation drugs, infections, Hyperhomocysteinemia (HHCY), antiphospholipid antibodies (aPL), and SLE may be closely associated with PCVT [7–12]. Several articles have described hereditary thrombophilia such as Factor V Leiden mutation (FVL), proteins C (PC) and protein S (PS), antithrombin III (ATIII) deficiency in PCVT [12–22]. However, these existing studies have not comprehensively analyzed the risk factors or etiology of PCVT, and the research is mainly conducted in European countries, India, and other countries, while there are a few studies in China which do not mention the acquired and genetic thrombophilia of PCVT, indicating a knowledge gap in this area [23–26].

For pregnant and puerperal women, early identification of risk factors associated to PCVT can reduce the incidence of PCVT. How do we identify women who are susceptible to PCVT, and what are the characteristics of these populations? On the other hand, what are the potential etiologic factors that should be a concern for women diagnosed with PCVT? Data in China is lacking. This study aims to investigate the risk factors of susceptible pregnant and postpartum women and the underlying causes of PCVT patients in a comprehensive hospital in China.

## Methods

### Study design and patient selection

This was a single-center retrospective cohort study. We consecutively included patients who were diagnosed with CVT during pregnancy or within six weeks after delivery from January 2009 to December 2022 registered in Peking Union Medical College Hospital (PUMCH). In the study, CVT was diagnosed according to established criteria and confirmed by at least one of the following neuroradiological examinations: computed tomography venography (CTV), magnetic resonance imaging (MRI), magnetic resonance venography (MRV), or digital subtraction angiography (DSA) [27]. Our institution followed certain basic principles for examinations selection. For pregnant women, doctors discussed the patients' clinical condition and the radiation risk of a head CT with the patients and their families (although studies have shown that the radiation dose of a head CT was below the teratogenic threshold for the fetus [28]). Given the sensitivity of CT diagnosis, head MRI and MRV were still the preferred examination. CTV scans were rarely performed at our center. For postpartum women, a rapid head CT was performed for initial assessment, followed by head MRI and MRV. DSA examination was considered only when the above-mentioned examinations fail to provide a clear diagnosis.

To explore the characteristics of pregnant and puerperal women susceptible to PCVT, we consecutively matched pregnant women in the first, second and third trimesters of pregnancy with matching gestational age in the obstetrics outpatient prenatal examination of our hospital, and hospitalized puerperium women without PCVT as controls in a 1:4 ratio. Women with these comorbidities were excluded from the selection of controls, including infertility or in vitro fertilization, pregnancy-induced hypertension or preeclampsia, underwent hormone therapy, had history of hemorrhage during labor or emergency delivery, malignancy, traumatic brain injuries, and incomplete data.

The study was approved by the local hospital Ethics Committee (JS-2420). Written and informed consent was obtained from all participants.

### Data collection

Demographic characteristics, medical history, clinical manifestations, and treatment for included patients were collected from the medical records. According to the current literature, the following laboratory results were intensively collected: routine blood count, coagulation, liver and kidney function, thrombophilia tests such as PC, PS, ATIII and activated PC resistance (APC-R), aPL such as anticardiolipin antibodies (aCL), anti-β2-glycoprotein I antibodies (aB2GPI), lupus anticoagulant (LA), and antinuclear antibodies, and homocysteine), imaging data (CT, MRI, MRV, DSA). Whole Exome Sequencing (WES) results were searched and summarized. Whether PCVT patients underwent WES screening was obtained from their medical records. Severity of illness related to symptoms of CVT was assessed by Glasgow Coma Scale (GCS) [29] and modified Rankin Scale (mRS) [30].

### Statistical analysis

Characteristics were presented as mean (standard deviation) for continuous variables and frequency (%) for categorical variables. Differences between subjects with and without PCVT were compared using the Mann–Whitney U-test for continuous variables and the Chi-square test for categorical variables. Statistical significance was defined as two-tailed *P* < 0.05, unless otherwise specified. All statistical analyses were performed using SPSS, version 23.0.

## Results

From January 2009 to December 2022, we identified 311 hospitalized patients diagnosed with CVT. The patients included 183 females and 128 males, with a female-to-male ratio of approximately 1.5:1. Among the female patients, 130 were of childbearing age (15–45 years), and 16 had CVT during pregnancy or postpartum.

Table 1 presented the demographic, clinical, and imaging characteristics of 16 CVT patients during pregnancy or postpartum. The average age was 29.06 ± 5.36 years. The majority of CVT events occurred in the first trimester (43.75%) and postpartum (37.5%). The most common symptom was headache (87.5%), followed by altered consciousness (50%), visual disturbances (50%), seizures (37.5%), and focal neurological deficits (37.5%). Tinnitus, cognitive impairment, and abnormal mental behavior were also be reported by included patients.

**Table 1:** Demographic and clinical characteristics of total patients

Characteristics	Total Patients (*n* = 16)
Demographics	
Age (mean ± SD)	29.06 ± 5.53
Phase, n (%)	
First trimester	7 (43.75)
Second Trimester	1 (6.25)
Third Trimester	0
puerperium	6 (37.5)
Post-abortion	2 (12.5)
Admission GCS score	
≥ 9	13
< 9	3
Clinical characteristics, n (%)	
Headache	14 (87.5)
Altered consciousness	8 (50)
Seizure	6 (37.5)
Visual impairment	8 (50)
Focal neurological deficits	6 (37.5)
Cognitive impairment	3 (18.75)
Mental abnormality	1 (6.25)
Tinnitus	3 (18.75)
Occluded sinus/vein, n (%)	
superior sagittal sinus	10 (62.5)
Transvers sinus	11 (68.75)
Sigmoid sinus	7 (43.75)
Inferior sagittal sinus	1 (6.25)
Cortical vein	3 (18.75)
Deep system (Straight sinus/VG/ICV)	2 (12.5)
Internal jugular vein	2 (12.5)
More than one sinus	11 (68.75)
Parenchymal lesion on imaging	
Hemorrhagic lesion	8/14
Non-hemorrhagic lesion (edema, infarction)	10/14
Anticoagulation treatment	15 (93.75)
mRS at discharge	
0–2	14
3–5	2
6	0

*Abbreviation: SD* standard deviation, *GCS* Glasgow coma scale, *mRS* modified Rankin Scale, *VG* vein of Galen, *ICV* internal cerebral vein

The most frequently involved venous sinus was the transverse sinus (68.75%), followed by the superior sagittal sinus (62.5%) and the sigmoid sinus (43.75%). Three patients had cortical vein involvement, with one patient suffering from isolated cortical vein thrombosis (Table 4, patient 3). This patient experienced headache, seizures, and visual impairment, and presented with brain edema and parenchymal hemorrhage. Eleven patients had multiple venous sinuses involvement.

To explore risk factors and clinical susceptibility to developing PCVT, we compared 16 PCVT patients with 64 pregnant and puerperal women without PCVT (Table 2). The frequency of anemia, thrombocytosis, and thrombocytopenia during pregnancy, pre-pregnancy anemia, and dehydration was significantly higher in people with PCVT than in those without PCVT (*P* < 0.05). Women with and without PCVT had no significant differences in age ≥ 35 years, frequency of pregnancy, gestational diabetes, pre-pregnancy diabetes, PCOS, concurrent fever or infection, or hematological indicators (*P* > 0.05). Although there were no significant differences between women with and without PCVT in obesity, history of adverse abortion, history of CVT, or use of ovarian stimulation drugs (*P* > 0.05), the proportion of those in PCVT patients was higher than controls. In addition, there were no woman combined with spontaneous low cranial pressure, jugular vein cannulation, past medical history of antiphospholipid syndrome (APS), SLE, venous thromboembolism (VTE), paroxysmal nocturnal hemoglobinuria, erythrocytosis, and nephrotic syndrome, or family history of VTE and CVT in both groups (not shown in Table 2). Due to the lack of data on thrombophilia and aPL for non-PCVT women, we did not make a relevant comparison.

**Table 2:** The risk factors of the study participants by pregnancy-related cerebral venous thrombosis

Characteristics	Pregnancy-related cerebral venous thrombosis		
	Yes(*n* = 16)	No(*n* = 64)	*P* value
Age ≥ 35 (y), n (%)	3 (18.8)	22 (34.4)	0.228
BMI > 30 (kg/m^2^), n (%)	2 (14.3)	5 (7.8)	0.801
Ever smoking, n	0	0	
First pregnancy/delivery, n (%)	6 (40)	31 (48.4)	0.556
History of adverse abortion, n (%)	2 (12.5)	3 (4.7)	0.260
History of CVT, n (%)	1 (6.3)	0	0.200
Pre-pregnancy anemia, n (%)	2 (12.5)	0	0.038
Pre-pregnancy diabetes, n (%)	0	1 (1.6)	1
PCOS, n (%)	0	3 (4.7)	1
Gestational diabetes, n (%)	1 (6.3)	9 (14.1)	0.673
Dehydration, n (%)	2 (12.5)	0	0.038
Concurrent fever/Infection, n (%)	0	3 (4.7)	1
Oral contraceptive use, n	0	0	
Ovarian stimulation drugs, n (%)	1 (6.3)	1 (1.6)	0.362
Anemia in pregnancy, n (%)	4 (25)	3 (4.7)	0.038
Thrombocytosis in pregnancy, n (%)	2 (12.5)	0	0.038
Thrombocytopenia in pregnancy, n (%)	5 (31.3)	0	< 0.001
Fibrinogen (g/L), mean (SD)	3.94 (1.47)	3.75 (0.75)	0.927
Neutrophil (10^9^/L), mean (SD)	7.00 (3.33)	7.24 (2.44)	0.665
Lymphocyte (10^9^/L), mean (SD)	1.79 (0.53)	1.67 (0.51)	0.459
Platelet to lymphocyte ratio, mean (SD)	130.80 (98.73)	139.05 (46.32)	0.683
Neutrophil to lymphocyte ratio, mean (SD)	4.27 (2.47)	4.93 (2.70)	0.354
Platelet (10^9^/L) × Neutrophil(10^9^/L) /Lymphocyte(10^9^/L), mean (SD)	834.70 (712.70)	1017.90 (529.82)	0.102

*Abbreviation*: *SD* standard deviation, *BMI* body mass index, *PCOS* polycystic ovary syndrome, *CVT* cerebral venous thrombosis

*P* value was for the test of difference between people with and without pregnancy-related cerebral venous thrombosis

Table 3 shows a detailed description of 11 PCVT patients without WES. Four of them were primiparous. Six of them had no pre-existing medical conditions before pregnancy. Two of them had a history of spontaneous abortion, two had a history of anemia, and one had a history of CVT. Although ***patient 1*** had normal aPL, she was suspected of seronegative APS based on the history of spontaneous abortion, thrombocytopenia, and CVT event. Patient 1 also had a protein S deficiency, which was not confirmed by genetic testing. ***Patient 3*** had hyperemesis gravidarum and thrombocytopenia during pregnancy, and hematology experts suggested follow-up for thrombocytopenia without treatment. ***Patient 4*** was obese and had a 10-year history of iron deficiency anemia, suffered from hyperemesis gravidarum, and was found to have HHCY during pregnancy. ***Patients 5 and 10*** were diagnosed with APS and SLE, respectively. ***Patient 6*** was obese and was found to have thrombocytosis during pregnancy. ***Patient 8*** had a recurrence of PCVT, with the first episode occurring in the fourth month of pregnancy accompanied by thrombocytopenia without a clear diagnosis and long-term management. Unfortunately, preventive anticoagulant therapy was not administered during this pregnancy. ***Patient 9*** had a history of using ovarian stimulation drugs, although this pregnancy was conceived naturally. ***Patient 11*** had a family history of primary thrombocytosis, and Janus Kinase 2 V617F mutation (JAK2 V617F) was detected during follow-up. Two patients did not have any other risk factors or etiologies besides pregnancy.

**Table 3:** Characteristics of patients without whole exome sequencing

	Age (y)	Phage	Pregnancy/childbirth history	PMH	BMI (kg/m^2^)	Platelet (10^9^/L)	D-D (mg/L FEU)	Thrombophilia	aPL	HCY (umol/L)	Risk factors, etiology	Pregnancy outcome
1	23	1st trimester (7th week)	G2P0	***Spontaneous abortion***	< 30	***34***	***765ug/L*** ***(0–420)***	***PS: 37% (L)*** APC-R: ND	N	9.7	Hereditary PS deficiency? Special type of APS?	induced abortion
2	19	1st trimester (3rd month)	G2P0	Healthy	26.16	249	***3.28***	APC-R: ND	N	14.9	/	induced abortion
3	28	1st trimester (8th week)	G1P0	Healthy	18.59	***42***	***20.62***	N	LA: ND	ND	Thrombocytopenia; Dehydration	induced abortion
4	34	2nd trimester (6th month)	G3P1	***IDA for 10yrs***	***30.49***	229	***2.08***	N	N	***42.9***	IDA; HHCY; Dehydration; Obesity	Cesarean section, stillbirth
5	35	1st trimester (6th week)	G7P0	***Recurrent miscarriage***	27.54	***65.7***	NA	NA	***ANA (* +*)*** ***aB2GPI (* +*)*** ***LA (* +*)***	NA	APS	induced abortion
6	34	Induced abortion (1 month)	G2P1	Healthy	***31.25***	***521***	0.5	N	LA: ND	6.1	Thrombocytosis Obesity	
7	20	Puerperium; cesarean section (4th day)	G1P1	Healthy	< 30	166	275ug/L (0–420)	N	aB2GPI: ND	ND	/	
8	24	1st trimester (8th week)	G2P0	***CVT, thrombocytopenia during last pregnancy***	25.64	**53**	***967ug/L*** ***(0–420)***	PC: 181% (H)	N	5.1	Thrombocytopenia	induced abortion
9^a^	34	Puerperium; cesarean section (11st day)	G1P1	Healthy	NA	326	***5.93***	N	ND	7.5	History of using ovarian stimulation drugs	
10	30	Spontaneous incomplete miscarriage (7th day)	G2P1	***Anemia*** ***for* > *10yrs***	21.48	***24***	***20.69***	***PS: 50% (L)*** PC: 199% (H) *ATIII: 74% (L)*	N	11.3	SLE (Hematological, kidney, multiple serous fluid accumulation, hypoprotein)	
11	34	1st trimester (6th week)	G1P0	Healthy	18.18	***550***	NA	***PS: 45% (L)***	N	12.4	Essential thrombocythemia	cesarean section; a healthy baby

*Abbreviation*: *PMH* past medical history, *L* low, *H* high, *N* normal, *ND* not detected, *NA* not available, *BMI* body mass index, *D-D* D-dimer, *aPL* antiphospholipid antibodies, *HCY* homocysteine, *PC* Protein C,* PS* Protein S, *ATIII* antithrombin III, *APC-R* activated protein C resistance, *ANA* anti-nuclear antibody, *aCL* anticardiolipin, *aB2GPI* anti-β2-glycoprotein I antibodies, *LA* lupus anticoagulant, *APS* antiphospholipid syndrome, *SLE* systemic lupus erythematosus, *IDA* iron deficient anemia

Thrombophilia test included PC, PS, ATIII and APC-R, and the result was normal unless otherwise specified

aPL included ANA, aCL, aB2GPI and LA, and the result was normal unless otherwise specified

^a^Recurrent hand joint pain and morning stiffness during pregnancy. Patient requested transfer and did not complete APS related examinations

Five PCVT patients hospitalized in the last few years were sent for WES. All of them had no past medical history before pregnancy. ***Patient 1*** had a significant PS deficiency, and WES showed a heterozygous PROS1 mutation. ***Patient 2*** was diagnosed with APS, and at the same time, PS decreased, but no PROS1 mutation was found in WES. Interestingly, there was a compound heterozygous F8 gene mutation for patient 2. ***Patient 3***, who was anti-BGPI positive, was diagnosed with APS during follow-up. ***Patient 4*** was diagnosed with APS with multiple aPL positive, and her PS level merely slightly decreased. There was homozygous MTHFR in patient 3 and patient 4. ***Patient 5*** was considered to have a hereditary PC deficiency due to heterozygous PROC mutation was found in WES (Table 4).

**Table 4:** Characteristics of patients performed whole exome sequencing

	Age (y)	Phage	Pregnancy/ childbirth history	PMH	BMI (kg/m^2^)	D-D	Thrombophilia	aPL	HCY	WES-Likely pathogenic	Risk factors, etiology
1	35	Puerperium (7th day)	G1P1	Healthy	27.51	**4.03**	***PS: 28% (L)***	N	10.8	***Heterozygous*** ***PROS1*** c.301C > T (p. Arg101Cys)	Hereditary PS deficiency
2^a^	25	Puerperium; Spontaneous labor (18th day)	G2P2	Healthy	22.76	**2.39**	***PS: 12% (L)***	***LA: 1.28(H)***	ND	***Heterozygous*** ***F8*** c.144-1259C > T ***Heterozygous*** ***F8*** c.6724G > A (p. Val2242Met)	APS; Hereditary thrombophilia?
3	32	Puerperium; Spontaneous labor (5th day)	NA	Healthy	NA	NA	N	***aB2GPI (* ±*)***	13.6	***MTHFR TT***	APS
4	32	Puerperium (7th day)	G1P1	Healthy	21.88	**3.76**	***PS: 62% (L)***	***ANA (* +*)*** ***ACL (* +*)*** ***aB2GPI (* +*)*** ***LA: 1.32(H)***	8.9	***MTHFR TT***	APS
5	26	1st trimester; induced abortion (11th week)	G2P1	Healthy	19.53	**1.95**	***PC: 39%(L)***	N	10	***Heterozygous*** ***PROC*** c.1218G > A (p. Met406Ile)	Hereditary PC deficiency

*Abbreviation*: *PMH* past medical history, *L* low, *H* high, *N* normal, *ND* not detected, *NA* not available, *BMI* body mass index, *D-D* D-dimer, *aPL* antiphospholipid antibodies, *HCY* homocysteine, *PC* Protein C, *PS* Protein S, *ANA* anti-nuclear antibody, *aCL* anticardiolipin, *aB2GPI* anti-β2-glycoprotein I antibodies, *LA* lupus anticoagulant, *MTHFR TT* Homozygous MTHFR c.677C > T (p. Ala222Val)

Thrombophilia test included PC, PS, ATIII and APC-R, and the result was normal, unless otherwise specified

aPL included ANA, aCL, aB2GPI and LA, and the result was normal, unless otherwise specified

^a^Combined pulmonary embolism and inferior vena cava embolism

WES-Uncertain significance: Patient 1: Heterozygous JAK2 c.2959G > A (p. Glu987Lys); Patient 3: Heterozygous MMACHC c.609G > A (p. Trp203*), Heterozygous TMEM199 c.2 T > C (p.0?), Heterozygous F13B c.986-15A > G; Patient 4: Heterozygous F13A1 c.1081G > A (p. Val361Met); Patient 5: Heterozygous F5 c.3331G > A (p. Ala1111Thr)

Among the 16 patients, five were diagnosed with APS, one was diagnosed with SLE, three had distinct protein S deficiency, one had protein C deficiency, six had anemia, five had thrombocytopenia, and two had thrombocytosis. Notably, except for one patient (Table 3, patient 1) was lost to follow-up, the remaining four patients underwent follow-up and obtained repetition of serology at least 12 weeks apart, confirming the diagnosis of APS. Except for one patient (Table 3, patient 3) who declined anticoagulation due to concerns about thrombocytopenia, all patients received anticoagulant therapy. Patient 11 received therapeutic doses of low molecular weight heparin (LMWH) until 6 weeks postpartum. Other seven patients who suffered from PCVT during pregnancy received unfractionated heparin (Table 3, patient 1) or therapeutic doses of LMWH before undergoing induced abortion or caesarean section. Procedures were performed 24 h after discontinuation of LMWH, followed by continued LMWH, gradually transitioning to warfarin. Patients who had PCVT during the puerperium received LMWH and then transitioned to warfarin. Except one patient (Table 4, patient 2) received a combination of LMWH and rivaroxaban due to combining pulmonary embolism and inferior vena cava embolism gradually transitioning to warfarin. Based on the etiology, primary disease control was administered, such as immunotherapy with glucocorticoids or immunosuppressants for APS patients.

## Discussion

CVT is a rare cerebral venous disorder, and pregnant and puerperium women are at increased risk. In the present study, we reported a small retrospective cohort of PCVT, exploring the risk factors and clinical predictors of PCVT and focusing on PCVT patients’ underlying causes. The characteristics of dehydration, pre-pregnancy anemia, and the presence of anemia, thrombocytopenia and thrombocytosis during pregnancy suggested a greater susceptibility to PCVT. For PCVT patients, autoimmune diseases, hereditary thrombophilia, and hematological disorders were common causes. Nearly half of 16 patients had no pre-existing medical conditions before pregnancy, and PCVT could be the first manifestation of other diseases.

Pregnant and postpartum women are a high-risk group for CVT. Extensive physiological, biochemical, and anatomical changes throughout normal pregnancy and puerperium result in hypercoagulability, hemodynamic alterations, and vascular damage [31]. Poor dietary intake, vomiting, excessive sweating, hypovolemia, and anemia could cause hemodynamic changes. Puerperal infections can induce vascular inflammation and systemic vasospasm [31, 32]. There is data indicated that maternal venous thrombosis in general is 13 times more frequent in the postpartum than in pregnancy [33]. ISCVT study has reported that the postpartum period is more susceptible to PCVT occurrences [5]. In our study, most patients developed CVT in the first trimester and postpartum period, which was inconsistent with previous research findings. A previous study had similar results and the bimodal distribution phenomenon was reasonable [34]. Firstly, increased levels of vWF, fibrinogen, and factor VIII may trigger venous thrombosis in particularly susceptible women during early pregnancy. Previous reports suggested that women who have had CVT early on in their pregnancy tend to have another underlying mechanism for their hypercoagulable state such as aPL [35]. Secondly, postpartum-related conditions, such as dehydration, and prolonged bed rest might lead to the second peak of PCVT incidence. On the other hand, the small number of patients in our study may lead to unrepresentative results.

Previous studies have reported that anemia was associated with CVT [36]. Thrombocytosis, possibly a performance of myeloproliferative neoplasms such as essential thrombocythemia, and thrombocytopenia, possibly a manifestation of APS, were associated with CVT [37–39]. A cohort covering Pakistan, Turkey, and Mexico showed that anemia and dehydration were the commonest obstetric risk factors identified of PCVT [40]. In our study, except for anemia during pregnancy, history of pre-pregnancy anemia was also a significant risk factor for PCVT. However, to best of our knowledge, there was no study on the association of thrombocytosis during pregnancy and PCVT. We first proposed that thrombocytosis and thrombocytopenia during pregnancy were risk factors of PCVT. How can we differentiate between gestational thrombocytopenia and thrombocytopenia that may indicate an underlying disease, such as APS and immune thrombocytopenia, resulting in adverse events during pregnancy? Previous literature [41] indicated that a platelet count < 80 × 10^9^/L may serve as a trigger to conduct further investigations for an alternative etiology, and a platelet count < 50 × 10^9^/L is generally considered less likely to be gestational thrombocytopenia. Besides, patients with gestational thrombocytopenia generally have no history of prior thrombocytopenia (except during pregnancy) and typically resolve spontaneously within 1–2 months after delivery. For pregnant and puerperal women without past medical history, anemia, thrombocytosis, and thrombocytopenia might be indicative of a higher risk of PCVT, even the warning of autoimmune diseases, myeloproliferative neoplasms, and other diseases [37–39]. It was necessary to pay more attention and even search for the causes.

Our study found that autoimmune disease such as APS, hereditary thrombophilia, and hematological disorders were common underlying causes of PCVT, different from the etiologic profiles years ago [7, 42]. The most common cause in the past was head or neck infections. Economic development, better control of infectious diseases, and a better understanding of the relationship between CVT and autoimmune diseases, hematological disorders and thrombophilia have contributed to this change.

Autoimmune diseases, especially APS, might cause CVT. CVT is a rare complication of APS, with a reported incidence of 0.7%, and on the other hand, APS accounts for a large proportion of about 6–17% of CVT cases [39]. Autoimmune diseases were the most common cause, accounting for about 27.8% of CVT cases in women of childbearing age in a previous study at our hospital [43]. The neurologic damages caused by APS is related to immune mediated vascular, inflammatory, and direct neuronal effects. Small/microvascular thrombosis and the direct action of aPL, lead to the destruction of the blood–brain barrier, triggering leukocyte adhesion and complement activation, further leading to neurotoxicity of cytokines and antibodies [39]. In the present study, about 31% (5/16) of PCVT were diagnosed with APS. Among those five patients, three were completely healthy before pregnancy, and the other two patients had no other symptoms except for spontaneous abortion, suggesting that PCVT could be the first manifestation of APS. The diagnosis of APS is challenging and requires professional immunology experts to combine thrombotic events, adverse pregnancy outcomes, childbirth history, and aPL for a comprehensive evaluation. Besides, there was one patient with SLE who presented with PCVT. The mechanisms for venous thrombosis in SLE extend beyond the commonly acknowledged role of aPL [44]. Factors such as vascular endothelial damage, procoagulant factors secreted by inflammatory cells, hypoalbuminemia, hyperglobulinemia, and organ damage such as lupus nephritis and thrombocytopenia caused by the activity of SLE itself are all high-risk factors for venous thrombosis [45]. In our study, aPL in the SLE patient were negative, but the disease of SLE itself was highly active, with concurrent hematologic (thrombocytopenia, PS deficiency), renal impairment (proteinuria), hypoalbuminemia, etc. We considered these factors to be the causes of CVT in this patient. All patients received immunotherapy to control the primary disease. This highlights the importance of screening for autoimmune diseases in PCVT patients, which is essential for treatment and long-term management.

Hereditary thrombophilia is an important etiology for CVT, accounting for approximately 34%-41% of cases in cohort studies [4]. The most common genetic risk factors for VTE in white individuals, such as FVL, prothrombin G20210A mutation, are rare in Asian populations, while PC, PS, and antithrombin deficiency are important for VTE in Asians [46]. PC may inactivate factors Va and VIIIa. Patients with homozygous or compound heterozygous subtypes may present with fulminant purpura in early life, while those with heterozygous subtypes may develop thromboembolism later in life. In our study, we identified one patient with severe PC deficiency, and WES revealed a heterozygous mutation in PROC gene (c.1218G > A, Met406Ile) (Table 4, patient 5). A previous study in Korea reported that Met406Ile was the second most common mutation in VTE patients and was absent in healthy controls [47]. Met406Ile has also been detected in a Chinese family with VTE [48]. The authors used homology modeling to demonstrate that the missense mutation (Met406lle) in PROC gene resulted in steric clashes and instability of PC structure, which could impair the normal physiological function of PC and cause hypercoagulability. In addition, 37.5% (6/16) of our patients showed reduced PS activity. Among them, three patients were considered to be gestational PS deficiency. Three patients had severe PS deficiency. One patient had a heterozygous mutation in PROS1 (c.301C > T, Arg101Cys) (Table 4, patient 1). This mutation has been reported in a case of PS deficiency associated with a partial loss of APC cofactor activity [49]. Another patient with low PS activity did not have any PROS1 mutation but had a compound heterozygous mutation in the F8 gene (c.144-1259C > T; c.6724G > A, Val2242Met) (Table 4, patient 2), which might cause X-linked thrombophilia due to factor VIII defect [50]. However, we did not perform functional testing to confirm this, which is a limitation and a goal for future research. We speculated that the low PS level may be a consequence of consumption due to the thrombotic process. One patient was suspected to have hereditary PS deficiency but did not undergo WES (Table 3, patient 1). There was no ATIII, FVL, or prothrombin G20210A mutation in our study. These results emphasize the importance of genetic testing for patients with suspected hereditary thrombophilia, specifically in cases where the underlying causes are obscure or when significant abnormalities are identified in thrombophilia tests, such as severe deficiencies in PC and PS.

HHCY was reported to be associated with an increased risk of postpartum CVT [8]. However, there was no corresponding HHCY in our patients, which may be attributed to regular vitamin supplementation during pregnancy. The mechanisms by which HHCY causes thrombosis may include endothelial cell toxicity, promotion of smooth muscle cell proliferation and intimal thickening, decreased production of NO and prostacyclin, increased platelet adhesion, and activation of FV. The MTHFR 677TT genotype is generally associated with a milder elevation in homocysteine. The relationship between the MTHFR C677T polymorphism and CVT remains controversial. A meta-analysis of 7 studies found that the MTHFR 677TT genotype could increase the risk of CVT by 2.3 times [51], while another meta-analysis suggested a lack of sufficient data to support the MTHFR 677TT genotype as a risk factor for CVT [52] and a review indicated that MTHFR had not a role as thrombotic venous and arterial risk factor [53].

Hematological disorders are also an important underlying cause of PCVT. JAK2 V617F could cause CVT [54]. In our study, we detected this mutation in a patient with essential thrombocythemia during follow-up (Table 1, patient 11), which suggested that genetic testing is necessary for patients with significant thrombocytosis.

Although PCVT is a rare disease, it poses a serious threat to maternal and fetal health. If relevant risk factors could be identified during pregnancy in time, the occurrence of PCVT might be reduced. Universal screening for thrombophilia in pregnant women is not justified, and selective screening should be conducted instead [55]. Women with a personal or family history of VTE may undergo genetic thrombophilia testing [55], and results should be carefully interpreted to guide pregnancy prevention. If there is a history of unexplained miscarriages, stillbirths, or thrombosis, it is advisable for pregnant women to undergo aPL profile. Thrombophilia screening is not recommended for asymptomatic women without a personal or family history of VTE. For pregnant and puerperium women with dehydration, pre-pregnancy anemia, or combined with anemia, thrombocytopenia, and thrombocytosis, who are more likely to suffer from PCVT, we need to closely monitor them and conduct further investigations if necessary. For example, for patients with a platelet count < 80 × 10^9^/L, we should be vigilant about the possibility of APS. On the other hand, for confirmed PCVT, it is essential to perform an aggressive etiology screen for autoimmune diseases. For diagnosed PCVT patients, genetic thrombophilia screening may be deferred during the acute phase of treatment with anticoagulants, considering that the prevalence of low PS levels during pregnancy may be present up to 100%, 20% of pregnant women may have low levels of antithrombin, and there is pregnancy-associated PC resistance [55]. Additionally, anticoagulant medications could affect protein S and other levels. Therefore, PCVT patients could be advised to undergo genetic thrombophilia screening after discontinuation of anticoagulant therapy, with a risk of CVT recurrence due to cessation of therapy. WES may be a better option, especially for PCVT patients with unknown etiology, as it is not influenced by pregnancy or anticoagulant medications, and its results can guide subsequent treatment and long-term anticoagulation strategies. Individualized recommendations should be made after discussing the cost–benefit issues with the patient and their family.

For the diagnostic approach to pregnant women suspected of PCVT, it should be based on the clinical condition, the diagnostic utility of the available modalities and the risks of each, never delaying the imaging when necessary. There are some concerns regarding CT scan imaging safety, although studies have shown that the radiation dose of a head CT is below the teratogenic threshold for the fetus, so non-contrast MRI is the typical first choice in pregnancy. It is often recommended to perform MRI at 1.5 T or 3 T, preferably after early pregnancy. For lactating women, there are no significant concerns regarding the choice of imaging modality. Even Iodinated contrast media or Gadolinium-based contrast media could be used [28]. Regarding to the treatment of confirmed PCVT, in addition to selecting the appropriate anticoagulation, primary disease control is necessary and the optimal duration of anticoagulants depends on the etiology. For pregnant PCVT patients considering continuing pregnancy, LMWH is recommended for anticoagulation until delivery, and continued for at least 6 weeks postpartum. No oral anticoagulation is approved during pregnancy. For lactating women with PCVT, LMWH is the preferred anticoagulant choice, while UFH and warfarin are also acceptable during lactation. Direct oral anticoagulants should be avoided [3]. If the etiology is unknown or related to mild hereditary thrombophilia, anticoagulant therapy should be continued for 6–12 months (e.g., heterozygous FVL and prothrombin G20210A mutation, factor VIII elevation). For those who suffered from more than two episodes of CVT or had a severe hereditary thrombophilia (e.g., homozygous FVL and prothrombin G20210A mutation, PC, PS, antithrombin deficiency, complex thrombophilia, APS), long-term anticoagulant therapy should be considered [27].

Our study has several limitations that should be acknowledged. This is a retrospective study, and information bias and missing data are inevitable. In addition, the small number of patients and the Chinese origins of all the participants limited the generalizability of our findings. Due to the limitation of the lack of thrombophilia and aPL data in controls, we failed to build a complete risk model that warrants further investigation in future studies with larger cohorts. Nevertheless, this study still provides valuable data for PCVT, especially in China.

## Conclusion

In conclusion, this study emphasizes the necessity of recognizing susceptible pregnant and puerperal women with PCVT and the need for etiological screening. The occurrence of anemia, thrombocytopenia and thrombocytosis during pregnancy, dehydration and pre-pregnancy anemia suggests a greater susceptibility to PCVT. Autoimmune diseases, hereditary thrombophilia, and hematological disorders are common causes for confirmed PCVT patients. It is essential to screen for underlying causes for PCVT, especially hereditary or acquired thrombophilia, which could provide a more comprehensive insight and play a crucial role in long-term management.

## Data Availability

The data that support the findings of this study are not openly available due to reasons of sensitivity and are available from the corresponding author upon reasonable request. Data are located in controlled access data storage at Peking Union Medical College Hospital.
